# Genome Analysis of Conserved Dehydrin Motifs in Vascular Plants

**DOI:** 10.3389/fpls.2017.00709

**Published:** 2017-05-04

**Authors:** Ahmad A. Malik, Michael Veltri, Kelly F. Boddington, Karamjeet K. Singh, Steffen P. Graether

**Affiliations:** Department of Molecular and Cellular Biology, University of Guelph, GuelphON, Canada

**Keywords:** dehydrin, phytozome, motif search, intrinsically disordered protein, abiotic stress, cold stress, drought stress, salinity

## Abstract

Dehydrins, a large family of abiotic stress proteins, are defined by the presence of a mostly conserved motif known as the K-segment, and may also contain two other conserved motifs known as the Y-segment and S-segment. Using the dehydrin literature, we developed a sequence motif definition of the K-segment, which we used to create a large dataset of dehydrin sequences by searching the Pfam00257 dehydrin dataset and the Phytozome 10 sequences of vascular plants. A comprehensive analysis of these sequences reveals that lysine residues are highly conserved in the K-segment, while the amino acid type is often conserved at other positions. Despite the Y-segment name, the central tyrosine is somewhat conserved, but can be substituted with two other small aromatic amino acids (phenylalanine or histidine). The S-segment contains a series of serine residues, but in some proteins is also preceded by a conserved LHR sequence. In many dehydrins containing all three of these motifs the S-segment is linked to the K-segment by a GXGGRRKK motif (where X can be any amino acid), suggesting a functional linkage between these two motifs. An analysis of the sequences shows that the dehydrin architecture and several biochemical properties (isoelectric point, molecular mass, and hydrophobicity score) are dependent on each other, and that some dehydrin architectures are overexpressed during certain abiotic stress, suggesting that they may be optimized for a specific abiotic stress while others are involved in all forms of dehydration stress (drought, cold, and salinity).

## Introduction

In addition to damage caused by viral infections (Bol et al., 1990) and insect herbivores (Price et al., 1980), plants must also survive abiotic stresses such as drought, cold, and salinity (Wang et al., 2003). These stresses can all be considered a form of dehydration because they cause a decrease in the amount of free liquid water that is available to the plant. A large group of proteins, known as the late embryogenesis abundant (LEA) proteins, provide protection against such abiotic stresses (Tunnacliffe and Wise, 2007; Battaglia et al., 2008; Hincha and Thalhammer, 2012). First discovered in cotton plants during seed development (Galau et al., 1986), LEA proteins are found in plants after late embryogenesis, an essential part of the seed maturation process during which self-induced dehydration takes place (Cuming, 1999). Proteins of one sub-family within the LEA family are named dehydration proteins (dehydrins, also known as group II or D11 LEA proteins) because of their overexpression during dehydration stress (Close et al., 1989). The presence of dehydrin transcripts is highly correlated with a plant’s ability to withstand abiotic stress (Kosová et al., 2007), showing that dehydrins likely play an important protective role, however, definitive characterization of their biochemical function(s) has remained somewhat elusive. Several *in vitro* studies have shown that dehydrins are able to protect enzymes, DNA, and membranes from freeze-thaw damage (Graether and Boddington, 2014), and lipids from oxidation by reactive oxygen species (Hara et al., 2003). All of these results suggest that dehydrins are able to carry out a large number of different protective functions in the plant. Given the multiple dehydrins and other LEA proteins that are found in many, if not all, plants, it is possible that there is a certain level of redundancy in protection that will make determining their *in vivo* biological and biochemical function a challenge.

The dehydrin sequence is highly hydrophilic and generally lacks cysteine or tryptophan amino acids (Close, 1996). Not surprisingly, these proteins belong to a structural family of proteins known as intrinsically disordered proteins (IDPs) (Tompa, 2002; Uversky, 2002a,b). When alone in solution, IDPs do not have a defined three-dimensional structure. Instead, they tend to be quite dynamic and can sample a large number of different structures. This is the case for dehydrins, where circular dichroism (Lisse et al., 1996; Hughes et al., 2013) and NMR (Lisse et al., 1996; Findlater and Graether, 2009; Szalainé Ágoston et al., 2011) studies have shown that they consist of mainly random coil secondary structure. However, in the presence of a ligand, some IDPs can gain structure (Wright and Dyson, 2009). In the case of dehydrins, the presence of a membrane surface has been shown to cause the protein to gain partial helical structure (Koag et al., 2003; Clarke et al., 2015).

Sequence analysis of the dehydrin has revealed the existence of three conserved motifs: the Y-, S-, and K-segments. The K-segment is a 15 amino acid long motif that, by definition, must be present in order for a protein to be called a dehydrin (Close, 1996), although a dehydrin lacking a K-segment has been recently described (Perdiguero et al., 2012). The K-segment has been said to resemble a class “A” amphipathic α-helix, but our structural studies suggest that it is only very weakly helical in the absence of ligand (Hughes and Graether, 2011; Atkinson et al., 2016). The S-segment is a variable length motif consisting of a tract of Ser residues (Close, 1996). It is a phosphorylation site and has been theorized to require a C-terminal acidic region in order to be phosphorylated (Mehta et al., 2009). The phosphorylated S-segment has been shown to cause dehydrin translocation from the cytoplasm to the nucleus (Goday et al., 1994), and also to increase the calcium binding capacity of the protein (Alsheikh et al., 2003). The Y-segment is a six-residue motif, generally stated as being DEYGNP (Close, 1996). It has been suggested that the Y-segment is a nucleotide binding site due to its sequence similarity to the nucleotide binding site of *Escherichia coli* chaperone protein GroES (Close, 1996), although this has not yet been experimentally proven. The regions between these defined segments are generally poorly conserved, and are known as ϕ-segments. These segments have been observed to contain histidine-rich (Hara et al., 2005) and lysine-rich motifs (Mouillon et al., 2008; Eriksson and Harryson, 2011), but are still generally regarded as nearly random sequences. The function of the ϕ-segment is unknown, though we have suggested that its very high flexibility could allow optimal orientation of the K-segments in order to interact with their targets (Hughes and Graether, 2011).

Dehydrins are sub-classified into five architectures depending on the presence and order of the three major conserved motifs (Close, 1996). The K-, Y-, and S-segments are used to place dehydrins into the Y_n_SK_n_, K_n_, SK_n_, K_n_S, or Y_n_K_n_ architectures. The subscript n indicates that the segment can be found multiple times. For the K-segment, n is often two, but can be up to 13 copies (Liu et al., 2012), whereas for the Y- and S-segments n is usually one or two copies in higher plants, but can be zero. These differences in the number of segments and the length of the ϕ-segments explain the large range of dehydrin sizes: from 10 kDa (Labhilili et al., 1995) to 70 kDa (Kim et al., 2012). With regard to the order of the various segments in the protein sequence, in general the Y-segment, when present, is usually located near the N-terminus and the S-segment is mostly located N-terminal to the K-segment. The exception is the K_n_S dehydrin architecture, which has been cloned from several citrus plants (Hara et al., 2003). In this case the S-segment is located after a K-segment at the C-terminal end of the protein.

As more dehydrins have been discovered, it has become evident that the canonical K-segment dehydrin sequence (EKKGIMDKIKEKLPG) is not completely conserved. For example, the *Vitis riparia* YSK_2_ dehydrin has two K-segments (Xiao and Nassuth, 2006), where neither K-segment (RKKGMKEKIKERIPG and QQKGMMEKIKEKLPG) is an exact match the canonical K-segment. Likewise, the canonical Y-segment sequence can show variability, and the S-segment is usually defined as a tract of 4–8 serine residues, although a potentially longer S-segment motif has also been suggested (Svensson et al., 2002). In addition, several papers have identified other potential dehydrin sequence motifs (Hara et al., 2005; Mouillon et al., 2006; Eriksson et al., 2011), but their prevalence among the larger dehydrin family is not always known. We therefore set out to perform a multiple genome analysis of the conservation of these known segments and to potentially search for new segments and architectures in the dehydrin family.

## Materials and Methods

### Dehydrin Sequences

**FIGURE 1:**
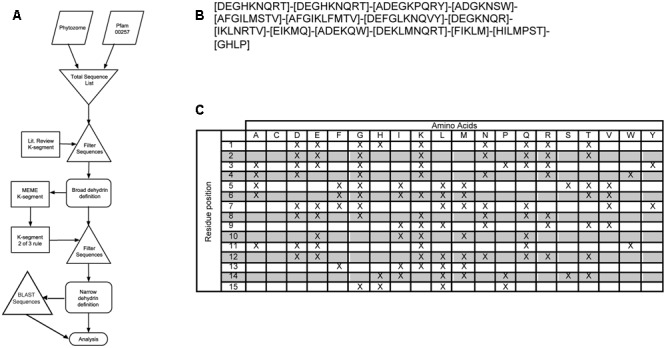
**Two-step filtering of dehydrin sequences. (A)** Flowchart of the selection process. Trapezoids represent sequence input from Phytozome 10 and Pfam00257 databases. Triangles represent the filtering of the sequences with the broad literature K-segment definition or the two-of-three rule as outlined in Section “Results,” while rectangles represent the filter search expression. Rounded rectangles represent data output. **(B)** Broad literature K-segment definition search expression. Square brackets show which residues may be present at that position while dashes show the separation of the residue positions. **(C)** Broad literature K-segment definition in table format. This contains the same information as in **(B)**, where ‘X’ indicates the possible presence of that amino acid at that residue position.

The Phytozome 10 dataset (Goodstein et al., 2012) of annotated protein sequences and the Pfam dehydrin sequence dataset (Pfam00257) were used as the sources for all dehydrin sequences. Pfam00257 entries were first filtered to remove duplicate sequences and sequences marked as fragments. Selection of the dehydrin sequence was performed as outlined in the flowchart (**Figure 1A**). By definition, a dehydrin must contain at least one K-segment. The initial K-segment pattern was based on a compilation of K-segment definitions from the literature (Supplementary Table S1). All Phytozome annotated protein sequences were filtered using a regular expression (search pattern) created during the literature search K-segment (**Figure 1B**), which represents a broad definition of the K-segment. To narrow the definition of dehydrins, the software program MEME (version 4.9.1) was run on the list of dehydrin sequences to create an unbiased narrower search expression for the K-segment (Supplementary Figure S1A) with the software parameters shown in Supplementary Table S4. This search expression was used to create a narrow dehydrin sequence dataset from the Phytozome sequences (Supplementary Table S5 and **File S1**) and Pfam00257 that was used for all subsequent analyses.

### Segment Sequence Analysis

The MEME program was used again as an unbiased approach to determine the Y- and S-segment sequences, and to potentially determine new motifs in the dehydrin family. The dehydrin sequence dataset was used in all searches except for species specific searches where only Phytozome 10 sequences were used. For all motif searches, the ‘any number of repeats’ mode was chosen. Parameters for the searches performed using MEME are shown in Supplementary Table S4, with all other parameters left at their default values. Results of the motif analyses were visualized using the LOGO format (Schneider and Stephens, 1990) and as position-weighted matrices. The statistical significance of discovered motifs is usually examined by calculating the *E*-value using MEME (McGrath et al., 2007). The *E*-value represents the expectation value of finding a motif with an equally conserved pattern in random sequences (Bailey et al., 2006). Because dehydrins, like many IDPs, have low sequence complexity regions, we repeated the MEME search using randomly shuffled dehydrin sequences using the program ‘shuffleseq’ (Rice et al., 2000), and calculated the *E*-value again.

For the GT-motif search, the motif width was varied from 5 to 20 in each individual MEME run. In addition to calculating the *E*-value, we determined whether the detection of the GT-motif occurred by chance by randomly shuffled the K_n_ dehydrin sequences enriched in the GT-motif before repeating the MEME search. The shuffling and search were performed five times.

The Shapiro–Wilk test for normality (Shapiro and Wilk, 1965) was calculated using ‘R’ (R Core Team, 2016) to determine if the ϕ-segment sequences are random. Amino acids were converted into the numbers 1–20 for the calculation. Sequences shorter than 30 residues were discarded from the test since they may result in false positives (Razali and Wah, 2011). The Shapiro–Wilk test was performed with the null hypothesis that the ϕ-segment followed a normal distribution. A normal quantile–quantile (Q–Q) plot was used to visualize the results.

We separately searched for dehydrins in the genomes of a lycophyte (*Selaginella moellendorffii*), non-vascular plants (*Marchantia polymorpha, Physcomitrella patens, Sphagnum fallax*), and green algae (*Chlamydomonas reinhardtii, Dunaliella salina, Coccomyxa subellipsoidea C-169, Micromonas pusilla CCMP1545, Micromonas* sp. *RCC299, Ostreococcus lucimarinus, Volvox carteri*) using BLASTP (Altschul et al., 1997) against known *P. patens* dehydrins (Ruibal et al., 2012), and searching with MAST (Bailey and Gribskov, 1998) using K-segment motifs found in all vascular plant dehydrins and discovered non-vascular plant dehydrins.

### Dehydrin Architecture and Biochemical Properties

Dehydrin sequences are modular in nature and as such the conserved Y-, S-, and K-segments could occur in any order and with different frequencies. To examine segment order in dehydrin architectures, a script was written to search for the various motifs and report the distances between them. To ensure an as broad as possible number of relevant hits, the two of three rule, as explained in Section “Results,” was used to search for K-segments (Supplementary Figure S1A), Y-segments (Supplementary Figure S1B) and S-segments (Supplementary Figure S1C). Dehydrin sequences were assigned to one of the five common architectures (K_n_, Y_n_SK_n_, Y_n_K_n_, SK_n_, or K_n_S). Novel and rare motifs were discovered using MEME (Bailey and Elkan, 1994) and GLAM2 (Frith et al., 2008).

Dehydrin sequences were submitted to the EXPASY server’s isoelectric point (pI) and molecular mass (M_r_) calculator to compute the theoretical pI and M_r_ (Gasteiger et al., 2005). GRAVY scores were calculated using the Sequence Manipulation Suite (Stothard, 2000). The molecular masses, pI values, GRAVY scores, and motif compositions were visualized in R using the bean plot package (Kampstra, 2008).

Patterns of dehydrin architecture were examined using microarray data from Genevestigator (Laule et al., 2008). Gene expression perturbations were examined during different abiotic stresses (drought, cold, and salinity), by plant anatomy and by developmental stage. The plant species examined were *Arabidopsis thaliana, Zea mays, Solanum lycopersicum, Oryza sativa, Medicago truncatula, Triticum aestivum*, and *Hordeum vulgare*. Affymetrix genome arrays were used for all species except for *Zea mays*, where the mRNA-seq Gene Level *Zea mays* platform was used. For species found in Phytozome 10, dehydrin genes were manually selected. For the remaining genomes the search term dehydrin was used to select genes, which were verified to match with our definition of a K-segment.

For gene upregulation during the abiotic stresses, the experimental data were obtained using the perturbation tool in Genevestigator. The data were then organized to exclude experiments that were performed in seeds since we are interested in protection in adult plants. The log2 fold change for all experiments were recorded at a significance level of α = 0.05, with log2 values of one or lower considered to be insignificant (i.e., assigned a value of zero). The data were arranged into groups based on the three perturbations being examined (i.e., drought, cold, and salinity). No down regulation was observed for any of the genes. The dehydrins from each organism were then organized based on their architecture, being grouped into K_n_, SK_n_, Y_n_SK_n_, or K_n_S. Insufficient data were available to examine the Y_n_K_n_ architecture. For localization of the different architectures, the gene expression of dehydrins in various anatomical regions of plants (seedling, inflorescence, shoots, roots, and, where available, callus) was examined. The log2 fold change for each experiment were recorded at a significance level of α = 0.05. For the developmental stages experiments, the specific developmental stages were grouped into ‘Early,’ ‘Middle,’ and ‘Late’ developmental stages as shown in Supplementary Table S5.

### Principal Component Analysis

CATPCA was performed using the SPSS Statistics (version 22) software package (2013). To prevent the averaging out of the biochemical properties, the variables pI, M_r_, and GRAVY score of each dehydrin were binned into low, medium and high ranges as defined in Supplementary Table S2. Architecture classification was based on the five major architectures found in the Phytozome dehydrins (K_n_, Y_n_SK_n_, Y_n_K_n_, and SK_n_). The presence of the SK-segment was treated as a separate variable. These properties were then tallied for each dehydrin in each species (Supplementary Table S3). After the CATPCA, three components were selected for further analysis on the basis that (1) the elbow in the Scree plot occurred at the third component, (2) the Eigenvalues were much greater than one (the fourth principal component had an Eigenvalue of 1.06 so it was cut) and (3) 68.3% of the variance is explained by the three components. Rotation was performed using the ‘varimax’ protocol (Kaiser, 1958). Coefficients with absolute values less than 0.3 were considered insignificant and therefore ignored in the subsequent analysis.

## Results

### Selection of the Dehydrin Protein Sequences

Thirty-five annotated vascular genomes were acquired from the Phytozome, version 10 (Goodstein et al., 2012). While this work focuses on dehydrins from higher (i.e., vascular) plants, an analysis of non-vascular plants is also performed. The Phytozome 10 file containing all protein-coding sequences, with alternative splice variants, was used as the source for the genome sequences, since a study on *Vitis riparia* dehydrins showed that alternative splicing can partially account for the sequence variation (Xiao and Nassuth, 2006). The Pfam dehydrin dataset (PF00257) is a collection of protein sequences that have been annotated or detected to be dehydrins (Finn et al., 2014). Duplicate sequences and fragment entries in the Pfam dataset were removed before further use.

Our first goal was to obtain a broad sample of K-segments in order to perform an unbiased motif search for sequence conservation of this motif. However, in order to obtain a pool of dehydrin sequences, we had to first find protein sequences that contain at least one K-segment. To circumvent this circular problem, the dehydrin sequences were collected and filtered over multiple steps (**Figure 1A**). In the first step, we obtained the definition of K-segments from a large number of papers to develop a very broad, very comprehensive regular expression pattern (**Figures 1B,C** and Supplementary Table S1). Note that this search expression does not give any weight to any of the positions, but merely notes the presence of a particular amino acid. This filter results in a very large list of putative dehydrins, including some matches that were not representative of the dehydrin K-segment. This broad set of dehydrin sequences was used as input for the Multiple Expectation maximization for Motif Elicitation (MEME) program to find a consensus sequence of the K-segment (Bailey and Elkan, 1994). This consensus sequence was used to develop a matching rule where a sequence segment must match two of the three rules shown in Supplementary Figure S1A in order to be called a K-segment. This type of search allows for substitutions, deletions, and insertions that a normal regular expression does not allow, but at the same time prevents a large number of false positives from occurring. This technique works under the assumption that the K-segment sequences being searched for are biologically functional and that the conserved core sections are responsible for this functionality. If we assume that the functionality is important *in vivo*, then proteins missing large parts of this motif would be considered questionable dehydrins and may need to be classified as dehydrin-like proteins instead. The result of this filtering step, 643 sequences, contains all proteins that we propose to be dehydrins. This narrow list was used for all subsequent analyses, including the motif searches.

### Analysis of Y-, S-, and K-Segment Sequences

**FIGURE 2:**
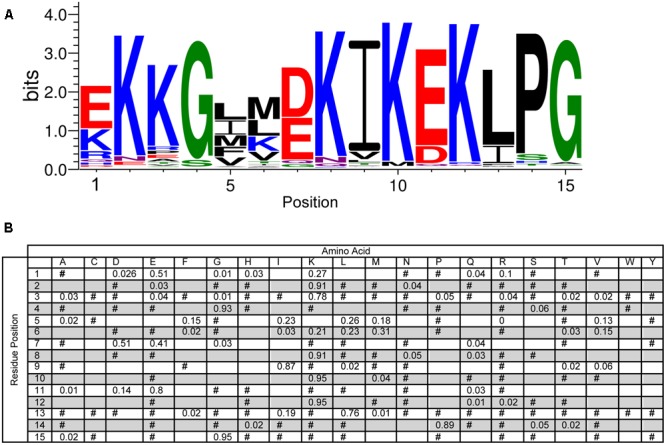
**Conservation of the K-segment sequence. (A)** LOGO representation of the MEME output of the K-segment. Amino acids are color-coded by their group type. Blue – positively charged (Lys, Arg, His); red – negatively charged (Asp, Glu); black – hydrophobic (Ala, Val, Leu, Ile, Pro, Phe, Met), green – polar (Gly, Ser, Thr, Tyr, Cys), purple – neutral (Asn, Gln). The heights of the amino acids correspond to their conservation at that position. Low probability amino acids may be too short to be seen. **(B)** Probability-weighted matrix (PWM) of the K-segment sequence. The probability of finding a particular amino acid at a particular position according to the search result. Empty space, *p* = 0; #, 0 < *p* < 0.01.

The narrow list of dehydrin sequences was used as input for the MEME program to allow for an unbiased search for the Y-, S-, and K-segments, and for the potential discovery of new motifs (Bailey and Elkan, 1994). A LOGO representation (Schneider and Stephens, 1990) of the MEME K-segment is shown in **Figure 2A**, while a position-weighted matrix (PWM) is shown in **Figure 2B**. As the prefix ‘K-’ suggests, this motif is rich in Lys amino acids, with the amino acid being highly conserved (>90%) at positions 2, 8, 10, and 12. Other highly conserved positions include a Gly at position 4 (93%) and a Pro-Gly at the end of the motif (89 and 95%). Several other positions are conserved in terms of the chemical properties of the amino acid: position 5, 9, and 13 are hydrophobic (95%), while positions 7 and 11 have the acidic residues Asp and/or Glu (>90%). The remaining positions are generally variable in terms of both amino acid type and property, although the N-terminal residues 1 and 3 are generally polar or charged, while position 6 is mostly hydrophobic.

**FIGURE 3:**
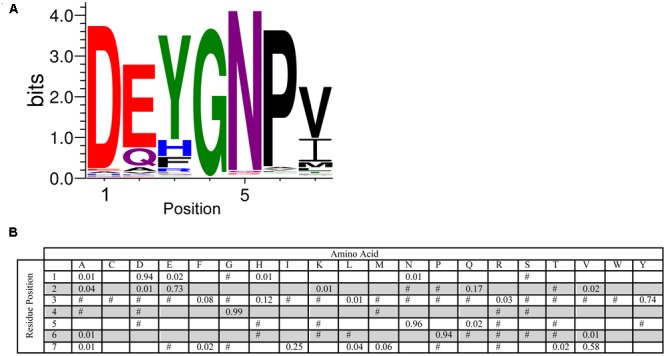
**Conservation of the Y-segment sequence. (A)** LOGO representation of the MEME output of the Y-segment. Amino acids are color-coded by their group type. Blue – positively charged (Lys, Arg, His); red – negatively charged (Asp, Glu); black – hydrophobic (Ala, Val, Leu, Ile, Pro, Phe, Met), green – polar (Gly, Ser, Thr, Tyr, Cys), purple – neutral (Asn, Gln). The heights of the amino acids correspond to their conservation at that position. Low probability amino acids may be too short to be seen. **(B)** PWM of the Y-segment sequence. The probability of finding a particular amino acid at a particular position according to the search result. Empty space, *p* = 0; #, 0 < *p* < 0.01.

The Y-segment is named after the presence of a Tyr amino acid in the middle of this motif. While tyrosine was a frequently found amino acid during the MEME search, alternate residues were also commonly found at this position. Tyr accounts for 74% of the amino acid present, while the next two most conserved amino acids are His (12%) and Phe (8%) (**Figure 3**). The presence of these three amino acids suggests that it is the aromatic character that is important at this position, although interestingly Trp was never detected. The most conserved group of residues is at positions 4–6, which consist of a Gly at position 4 (99%), an Asn at position 5 (96%) and a Pro at position 6 (94%). The first residue in the Y-segment is often Asp (94%), and is often followed by another acidic amino acid (Glu, 73%). In the literature, the Y-segment is often defined as being six residues long, however, as **Figure 3** shows, this motif ends with a hydrophobic amino acid at the 7th position 96% of the time.

**FIGURE 4:**
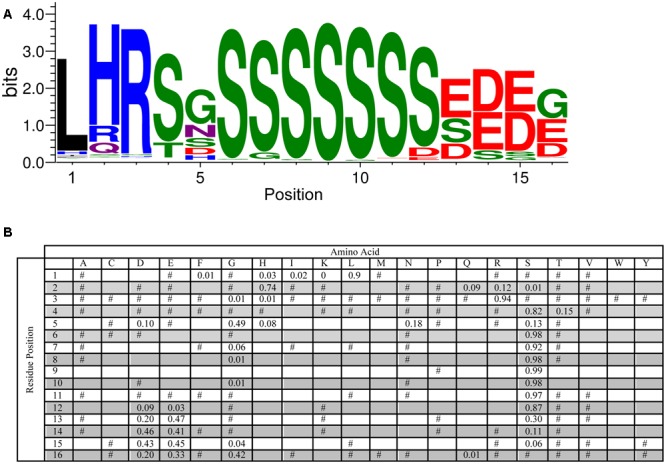
**Conservation of the S-segment sequence. (A)** LOGO representation of the MEME output of the S-segment. Amino acids are color-coded by their group type. Blue – positively charged (Lys, Arg, His); red – negatively charged (Asp, Glu); black – hydrophobic (Ala, Val, Leu, Ile, Pro, Phe, Met), green – polar (Gly, Ser, Thr, Tyr, Cys), purple – neutral (Asn, Gln). The heights of the amino acids correspond to their conservation at that position. Low probability amino acids may be too short to be seen. **(B)** PWM of the S-segment sequence. The probability of finding a particular amino acid at a particular position according to the search result. Empty space, *p* = 0; #, 0 < *p* < 0.01.

An analysis of the S-segment motif required a slightly different approach since the length of the Ser-tract is variable (though generally agreed to be 4–8 Ser residues long), and another work has reported the conservation of residues N-terminal to the Ser-tract (Svensson et al., 2002). To examine these two aspects, we performed the MEME searches with widths of 5–20 residues, and found that 16 residues was the optimum value (i.e., most conserved length) for the longer S-segment. As can be seen in **Figure 4**, the highest probability length is six serines. At the C-terminus of this tract a pair of Asp and/or Glu residues often follows the Ser-tract, and at the N-terminus there is often a Ser-Gly pair, which can be seen in the LOGO representation at positions 4–5 and 6–7. Even further toward the N-terminus the motif Leu-His-Arg (90, 74, and 94%, respectively), followed by Ser or Thr (82 or 15%) is found. These N-terminal residues show that Leu needs to be followed by a positively charged amino acid (His and Arg), and then a hydroxyl group containing amino acid (Ser or Thr). The fifth position has a preference for Gly (49%), but could be any non-hydrophobic amino acid.

We examined the *E*-values of the Y-, S-, and K-segments discovered by MEME, and compared them to *E*-values calculated from randomly shuffled dehydrin sequences using the same MEME run parameters (**Table 1**). As can be seen, the segment *E*-values are many fold higher, strongly showing the discovered motifs are statistically significant.

**Table 1:** *E*-value of the top motifs discovered with MEME on dehydrin and shuffled dehydrin sequences.

	*E*-value	*E*-value (shuffled)
K-segment	1.4 × 10^-13590^	9.6 × 10^-173^
S-segment	2.3 × 10^-4434^	2.1 × 10^-75^
Y-segment	1.8 × 10^-2012^	5.1 × 10^-41^
GT-motif	6.8 × 10^-3498^	8.0 × 10^-200^

*Run parameters are shown in Supplementary Table S4.*

### Analysis of the ϕ-Segment Sequence

**FIGURE 5:**
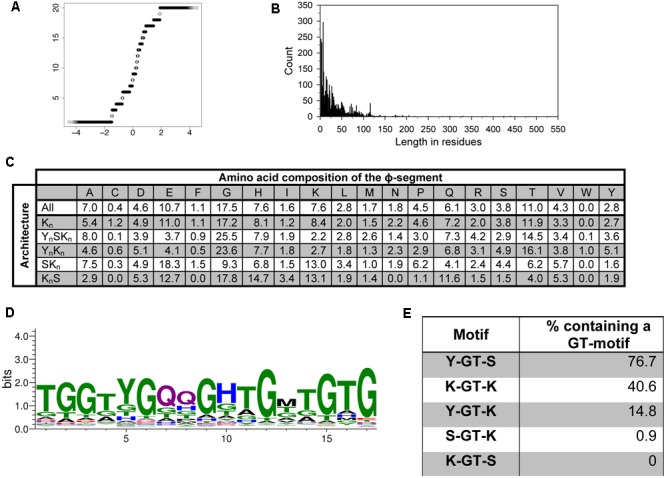
**The ϕ-segment sequence is not random. (A)** Q–Q plot of the ϕ-segment sequences. **(B)** Length in residues of the ϕ-segment as a histogram plot. **(C)** Overall amino acid composition of the ϕ-segment by architecture. The values indicate the percent of all residues that are that particular amino acid in the ϕ-segment. The composition is shown for all dehydrins and for each individual architecture. **(D)** LOGO representation of the GT-motif. Blue – positively charged (Lys, Arg, His); red – negatively charged (Asp, Glu); black – hydrophobic (Ala, Val, Leu, Ile, Pro, Phe, Met), green – polar (Gly, Ser, Thr, Tyr, Cys), purple – neutral (Asn, Gln). **(E)** Location of the GT-motifs between the other three segments expressed as percent occurrence for that segment pair.

To test whether the ϕ-segment is a random sequence, we took all sequences located between the Y-, S-, and K-segments to perform the Shapiro–Wilk test for normality (Shapiro and Wilk, 1965). A Q–Q plot can be used to visualize the data; deviation from a diagonal line would suggest that the data are not normally distributed (Wilk and Gnanadesikan, 1968). The Q–Q plot for the ϕ-segments is presented in **Figure 5A**, and shows that the sequence composition of the ϕ-segments is not random. We also looked at the distribution of the lengths of the ϕ-segment. **Figure 5B** shows a highly left-skewed distribution. This is partly caused by the large number of ϕ-segments that are located at the end of a protein after a K-segment, which tend to be very short (1–10 residues). The majority of the ϕ-segments (95%) are <100 residues in length, but can possibly extend out to several hundred residues for the larger dehydrins.

The ϕ-segments are often said to be rich in Gly, Ala, Ser, and Thr amino acids, but an examination of a large number of ϕ-segments shows that this is not completely correct in a larger dataset (**Figure 5C**). The top three amino acids in all ϕ-segment sequences are Gly (17.5%), Thr (11%), and Glu (10.7%). Other amino acids which occur with >5% frequency are Ala (7.0%), His (7.6%), and Gln (6%). The bottom three amino acids are Trp (0.05%), Cys (0.4%), and Phe (1.1%). All other amino acids occurred with frequencies between 1.6 and 4.6%. We also examined the ϕ-segment amino acid composition in the five different dehydrin architectures. For most amino acids, there is only a moderate difference between the architectures (**Figure 5C**). Exceptions are the more abundant amino acids such as Gly, Glu, and Lys. For the most part these can be differentiated by the presence of the Y-segment, such that the Y_n_SK_n_ and Y_n_K_n_ dehydrins contain higher amounts of Gly (24% versus 17% on average) but lower amounts of Glu (4% versus 15% on average) and Lys (2.5% versus 10% on average). The K_n_S dehydrins tend to be higher in His (14.7% versus 7.6% on average), Gln (11.6% versus 6.1% on average), and Lys (13.1% versus 7.6% on average).

We also performed an extensive motif discovery search on the ϕ-segment sequences to see if any recurrent motifs can be found that were previously missed. Individual MEME runs were performed on the full dehydrin sequences with the widths varied between 5 and 20 residues in each search, and a single run with the width allowed to vary between 8 and 50 residues. Only one novel motif was consistently observed, which is a GT-rich motif with an optimal width of 17 residues (**Figure 5D**). This sequence is very rich in Gly and Thr (dominating in 41 and 29% of the residue positions), and may contain two Gln residues in the middle.

The *E*-value of unshuffled and shuffled GT-motif searches is reported in **Table 1**. Once again, the unshuffled search has a much lower statistical probability of having occurred by chance. Nevertheless, the top hit in all of the shuffled sequence searches returned Gly-rich motifs (data not shown). To examine whether this sequence has occurred simply by chance due to the high Gly and Thr content in ϕ-segments, we took 236 K_n_ dehydrin sequences that are rich in GT-motifs and repeated the MEME search using shuffled sequences. The search was repeated using five different sets of shuffled sequences. No GT-motifs were detected, suggesting that this motif did not occur by chance.

We also examined between which Y-, S-, and K-segments the GT-motif is found. **Figure 5E** shows that GT-motifs are especially prevalent between Y- and S-segments and between K- and K-segments. They are infrequently found between Y- and K-segments and rarely found between S- and K-segments and were never found between K- and S-segments. With regards to dehydrin architecture, the GT-motif was found in all Y_n_SK_n_ architectures and in 82% of all Y_n_K_n_ architectures, suggesting that the presence of the Y-segment is a strong indicator for the presence of this motif. It is also found in 50% of the K_n_ proteins, nearly 40% of the K_n_S proteins, and approximately 25% of all of the SK_n_ dehydrins (**Table 2**). To determine whether these GT-motifs have a specific function or are merely a remnant of dehydrin evolution will require further evaluation.

**Table 2:** Presence of novel and rare motifs in the five architectures.

	K_n_	Y_n_SK_n_	Y_n_K_n_	SK_n_	K_n_S
All	98	254	45	216	33
GT-motif	50	254	37	52	13
SK-segment	n/a	217	n/a	37	n/a
Lys-rich	7	6	6	154	33
His-rich	1	3	0	7	0

There have been reports on the presence of Lys-rich (Mouillon et al., 2006; Eriksson and Harryson, 2011) and His-rich (Hara et al., 2005) motifs in dehydrins. For our purposes, we define the motif as being rich in that particular amino acid if it contains four or more contiguous His or Lys residues. A search of the dehydrin sequences revealed very few His-rich sequences (**Table 2**). The Lys-rich sequences are rare in K_n_, Y_n_SK_n_, and Y_n_K_n_ architectures (less than 15%), but occur in all K_n_S dehydrins and are present 71% of the time in SK_n_ dehydrins (**Table 2**). In the literature the Lys-rich segments have sometimes been labeled as K-segments, but the lack of hydrophobic residues in alternating positions with Lys would suggest that they are different form the K-segment, and most likely have a different function in dehydrins.

### Dehydrin Architectures

**FIGURE 6:**
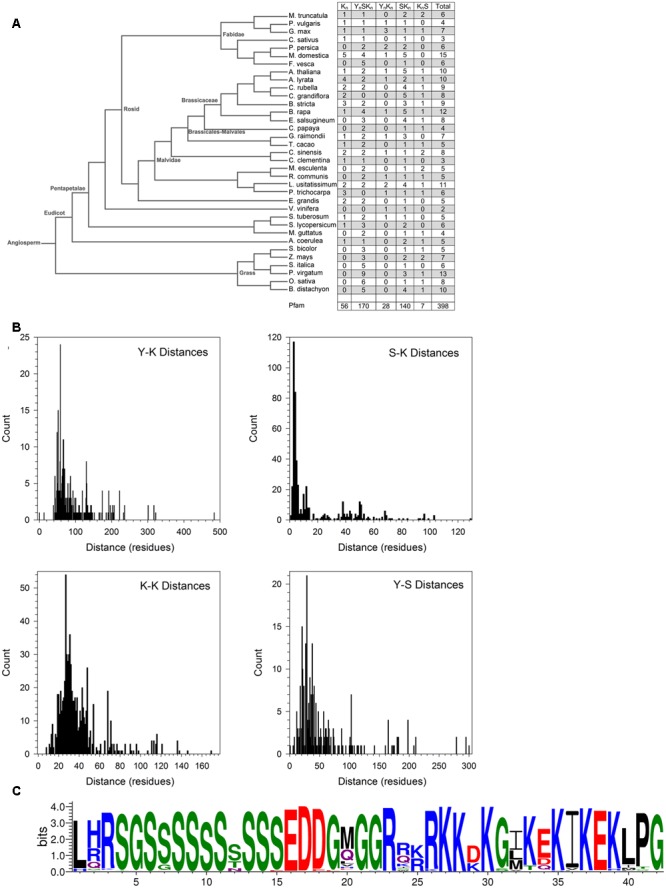
**Dehydrin architecture and discovery of the SK-segment. (A)** A phylogenetic tree of from Phytozome 10 of the species used in this study. The right column indicates the count of the architectures found in each species. **(B)** Distances in residues between the Y-, S-, and K-segments. The segment pairs are indicated inside each plot. **(C)** The SK-segment represented in LOGO form. Blue – positively charged (Lys, Arg, His); red – negatively charged (Asp, Glu); black – hydrophobic (Ala, Val, Leu, Ile, Pro, Phe, Met), green – polar (Gly, Ser, Thr, Tyr, Cys), purple – neutral (Asn, Gln).

The conserved Y-, S-, and K-segments in dehydrins are modular in nature and can be found with different frequencies (including zero) (Close, 1996). The five commonly listed architectures are K_n_, Y_n_SK_n_, SK_n_, Y_n_K_n_, and K_n_S. We counted the number of these different architectures in different species in Phytozome 10 and in Pfam 00257 (**Figure 6A**). Searches for the motifs were performed using the segment definitions found in Supplementary Figure S1. The last column in the figure lists the total number of dehydrins discovered in each species and in Pfam. An examination of the distribution by species reveals different preferences for some of the architectures between three groups (grasses, non-rosid dicots, and rosid dicots). Among the grasses, the S-segment appears quite prevalent since only Y_n_SK_n_, SK_n_, and K_n_S architectures are found. Among the non-rosid dicots, the K_n_ and Y_n_K_n_ architectures are somewhat rare but less so than in the grasses, while for rosids all of the four major architectures are seen. The SK_n_, K_n_S, and Y_n_SK_n_ architectures show no preference among the different species.

**FIGURE 7:**
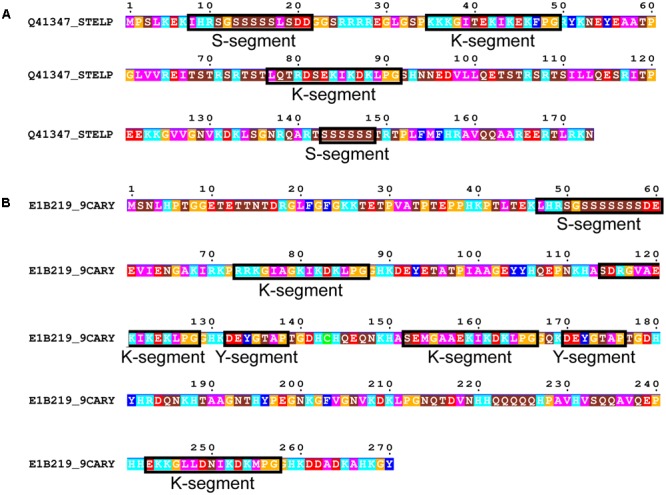
**Rare dehydrin architectures. (A)** SKKS dehydrin from *Stellaria longipes*. **(B)** SKKYKYK dehydrin from *Cerastium arcticum.* In both panels residues are colored according to: acidic residues, red; basic residues, light blue; aromatic residues, dark blue; hydrophobic residues, magenta; polar residues, brown; Pro or Gly, orange; Cys, green. Conserved motifs are boxed and labeled.

The order of the three major dehydrin motifs and the distribution of the distances between them were examined next. Even in this large dehydrin dataset, the order of the three different segments is highly conserved with only very few exceptions (see **Figure 7**). That is, the Y-segment is the most N-terminal motif, the S-segment is next, and the K-segment(s) occur at the C-terminal end of the protein. With regards to distances (**Figure 6B**), the median distances between the Y- and S-segments and between two K-segments is similar (∼30–40 residues), while the median distance between Y- and K-segments is longer at 70 residues and, with two exceptions, never shorter than 30 residues. We observed that the distances between S- and K-segments are unusually left-skewed to short distances in comparison to the others, with 3–6 residues occurring 56% of the time. To investigate this more closely, dehydrin sequences with short distances were extracted and examined with the gapped motif finder GLAM2 (Frith et al., 2008). The LOGO representation of the highest scoring sequence is shown in **Figure 6C**. This motif, which we have named as the “SK-segment,” is 40–42 residues long, and is a combination of the S-segment and K-segment with a short linker. The S-segment portion runs from position 1 to approximately 18, while the K-segment runs from approximately positions 27–42. Variability in these lengths is due to the variability in the gap, that is, in determining the end of the S-segment and the beginning of the K-segment. The gap, essentially the linker between the S- and K-segments, has some conservation of its own. The first part consists of Gly-Xaa-Gly-Gly-Arg (where Xaa is any amino acid), while the second part consists of Arg-[Arg, Glu, Lys]-[Lys, Arg]-Arg-Lys, where the square brackets denote alternative choices at that position. A search for the SK-segment among the different species found in Phytozome 10 did not show any distinct distribution pattern (Supplementary Figure S2). The SK-segment (**Table 2**) is found predominantly in the Y_n_SK_n_ architecture (85%) and sometimes in the SK_n_ architecture (17%).

We subsequently searched in our dehydrin sequence dataset for architectures that did not follow the typical order of Y-, S-, and K-segments. Only two dehydrins were found that could not be classified under one of the five common architectures. This includes a SKKS dehydrin from *Stellaria longipes* (**Figure 7A**) (Zhang et al., 1993) and a SKKYKYK dehydrin from *Cerastium arcticum* (Kim et al., 2013) (**Figure 7B**). The SKKS architecture consists of SK-K-S motifs, where the second S-segment is of the short form. In the SKKYKY dehydrin, the single S-segment is of the long form. The two Y-segments both contain Tyr, but the seventh residue is not hydrophobic (Thr in both cases), while several of the K-segments are not well-conserved because of a lack of Lys residues in the N-terminal part of this motif.

### Properties of Dehydrins and Their Protective Roles

**FIGURE 8:**
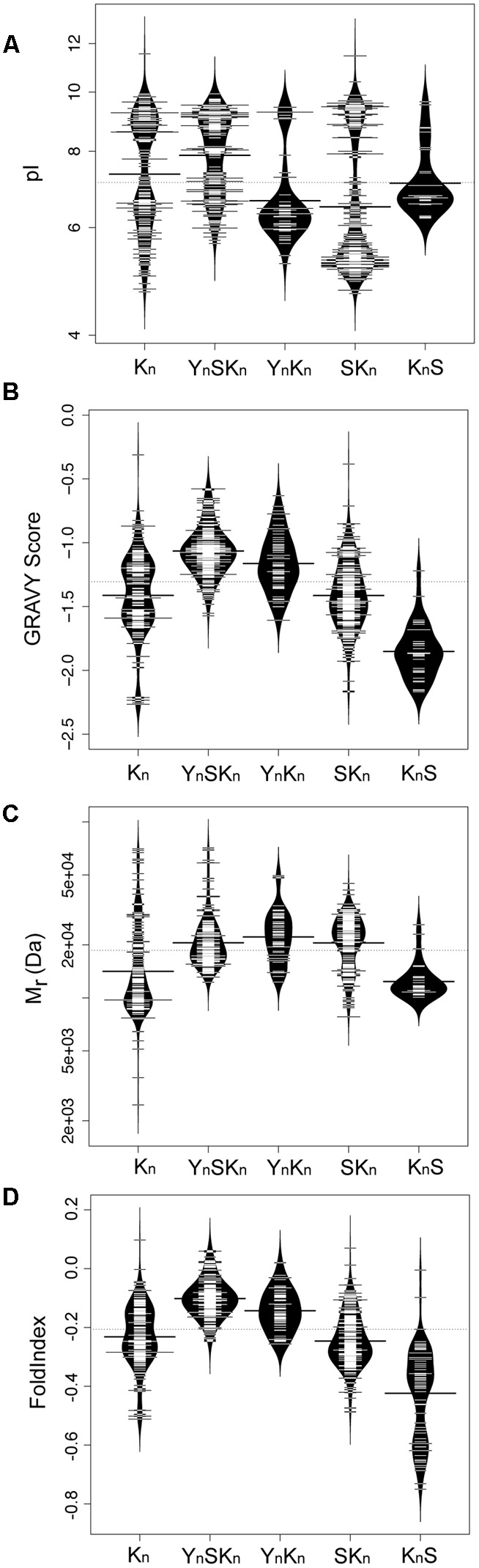
**Biochemical properties of dehydrins have unimodal and bimodal distributions.** Bean plots of **(A)** isoelectric point (pI), **(B)** GRAVY score, **(C)** molecular mass (M_r_), and **(D)** FoldIndex score of dehydrins categorized by the five architectures (K_n_, Y_n_SK_n_, SK_n_, Y_n_K_n_, and K_n_S). The thin bars represent an individual protein while the wide black bar represents the mean of each group. The violin shapes represent the density of values. The dotted line represents the mean value of all dehydrins over all of the architectures. The y-axes of the GRAVY and FoldIndex scores are linear scales while the M_r_ and pI are logarithmic scales.

We subsequently examined how some biochemical properties are affected by the dehydrin architecture. These biochemical properties include their molecular mass (M_r_, a measure of their size), isoelectric point (pI, a measure of their net charge), GRAVY score (a measure of their net hydrophobicity), and FoldIndex (propensity of a protein to fold) (Prilusky et al., 2005) which are shown in **Figure 8** as bean plots (Kampstra, 2008). The distribution of the pI scores is bimodal for four architectures (K_n_, Y_n_SK_n_, YnK_n_, and SK_n_), where all of them show a basic pI value centered around pH 9 (**Figure 8A**). The exception to this, the KnS architecture, consisted mainly of dehydrins with a near neutral pI and a few with a basic pI. The bimodal properties are interesting; unimodality would suggest a random sequence selection of that property while bimodality suggests that the proteins specifically evolved these properties, possibly in reference to their specific function. In the acidic range there is dissimilarity among the pI values of the different architectures; the K_n_ and Y_n_SK_n_ architectures show a wide range of values (between pH 5.0–7.0 and 6.0–7.0, respectively). Two other architectures show a narrower acidic pI range, with Y_n_K_n_ dehydrins having a center near pH 6 while the SK_n_ dehydrins have a center near pH 5, while the K_n_S dehydrins have an average pI near pH 7. For the GRAVY scores (**Figure 8B**), the K_n_ architecture shows a bimodal distribution, with the centers near -1.6 and -1.3. The four other architectures showed unimodal distributions. In the case of Y_n_SK_n_ there is a preponderance of dehydrins with GRAVY scores around -1. For Y_n_K_n_ and SK_n_, there is a large spread of GRAVY scores, centered at -1.2 for Y_n_K_n_, -1.4 for SK_n_, and -1.9 for K_n_S. The molecular mass plot (**Figure 8C**) did not show any bimodal distribution, but instead had a region where a majority of the M_r_s were found. For K_n_ and K_n_S this is around 12 kDa, while for the other three architectures it is around 20 kDa. Even with these average values it should be noted that there is a very large range of molecular masses for the dehydrins. With FoldIndex, negative scores represent proteins that are predicted to be unlikely to fold, and hence be intrinsically disordered. For the most part, the distribution of the FoldIndex scores (**Figure 8D**) follow that of the GRAVY scores (**Figure 8C**), suggesting that the disorder in dehydrins is driven by hydrophobic residues rather than the balance of charged residues. One exception to this pattern is K_n_S; the FoldIndex scores are spread over a large range of values, suggesting that in this architecture differences in the number of charged residues has an effect on the potential amount of disorder.

**FIGURE 9:**
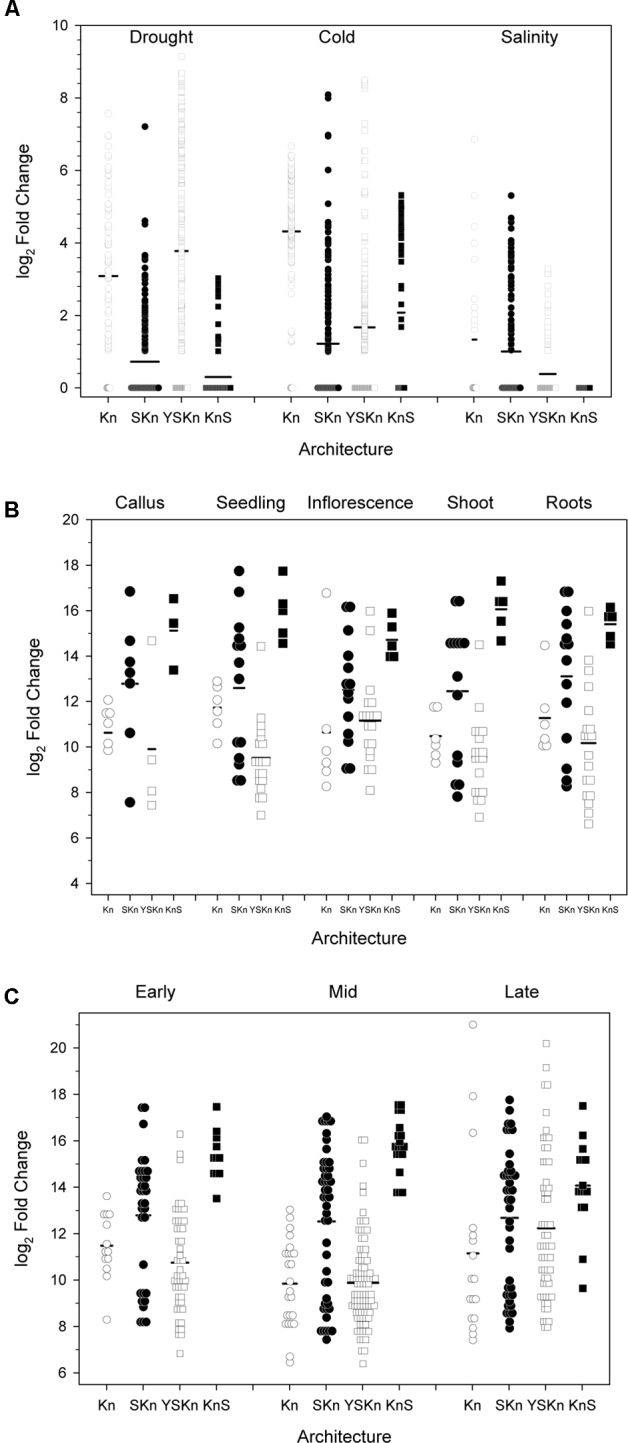
**Changes in dehydrin gene expression under various conditions.** The changes in gene expression are plotted as log2 changes based on the various dehydrin architectures. Symbols represent the results of an individual experiment, while the thick black bars are the average-fold change. Experiments showing a log2 fold change of less than one are shown as zero, with data points shown as overlapped symbols. **(A)** Abiotic stress, **(B)** Anatomy, **(C)** Developmental stage. K_n_, open circles; SK_n_, closed circles; YSK_n_, open squares; K_n_S, closed squares. Insufficient data were available to plot the YK_n_ architecture data.

We examined the change in expression of the different architectures under different stress conditions, in different tissues, and at different life stages (**Figure 9**). Details on how the data points were collected is described in Section “Materials and Methods.” Expression levels showing log2 values <1 are shown as being zero-fold change. An examination of the expression of the various dehydrins sorted by drought, cold, and salinity stress suggests that there is some preference for architecture by stress (**Figure 9A**). The K_n_ dehydrins are mostly upregulated during cold and drought. Under all stress conditions examined, the SK_n_ dehydrins showed on average very little change in expression. The Y_n_SK_n_ dehydrins are most upregulated during drought, but were only weakly upregulated during cold and salinity stresses. The K_n_S dehydrin shows the most upregulation during cold stress, minimally with drought stress, but not with salinity.

Examination of the relationship between architecture and anatomy (**Figure 9B**) or developmental stage (**Figure 9C**) did not show any substantial change for any of the architectures. In all cases, the log2 changes in expression levels were similar across all conditions.

The plotting of architectures versus these properties is one way to look for relationships among these proteins, but many other relationships are possible (in fact between each variable) and pairwise plots cannot show multiple relationships at the same time. We evaluated such potential relationships using a principal component analysis (Hotelling, 1933), specifically categorical principal component analysis (CATPCA) (Linting et al., 2007). The advantages of CATPCA are that it does not require assumptions of normality or linearity in the variables, and can be used in the analysis of categorical data such as dehydrin architecture. CATPCA also allows us to see what associations may exist between the five variables (pI, M_r_, GRAVY score, and architecture, to which we added the SK-segment motif). To prevent an averaging out of the values, properties are classified according to the ranges shown in Supplementary Table S2 and as described in Section “Materials and Methods.” The resulting CATPCA variable inputs are shown in Supplementary Table S3.

The variable coefficients after CATPCA are shown in **Table 3** where they are broken down by their contribution to the first three principal components (PC1-3). PC1 is dominated by the SK_n_ and K_n_ architectures (i.e., in the absence of a Y-segment), and is highly linked with acidic pI and medium M_r_ proteins. The GRAVY scores in PC1 are low, showing that these dehydrins are highly hydrophilic even in this family of disordered proteins. PC2 is dominated by the Y_n_SK_n_ architecture, and is linked with dehydrins that have high M_r_, higher GRAVY scores and basic pI. PC3 is associated with the SK_n_ architecture, and has a strong linkage with dehydrins with neutral pI. The K_n_S architecture has no notable association with PC1 or PC2, but has a strong negative association with PC3, showing that this architecture is not associated with a neutral pI.

**Table 3:** Coefficients of the Principal Components show a strong clustering of variables.

	Principal component		
	1	2	3
Arch Kn	0.828		
Arch YnSKn	0.392	0.859	
Arch YnKn		0.310	
Arch SKn	0.752		0.611
Arch KnS			–0.774
Motif SK		0.696	–0.343
Acidic pI	0.933	–0.643	
Neutral pI			0.747
Basic pI	0.517	0.884	
Low MW	0.683		
Medium MW	0.931	–0.619	
High MW	0.437	0.769	–0.330
Low GRAVY	0.912	–0.574	
Medium GRAVY	0.703	0.442	0.417
High GRAVY	0.495	0.753	

*Values are classified in Section “Materials and Methods”. Coefficients ≤| 0.30 | are not shown.*

### Dehydrins in Non-vascular Plants and a Lycophyte

**FIGURE 10:**
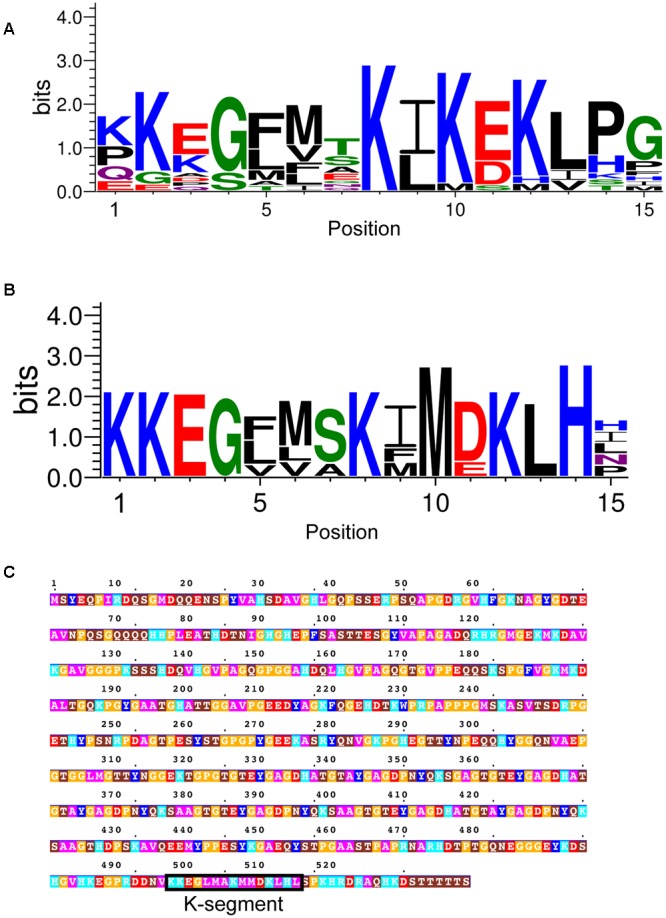
**Dehydrins in non-vascular plants and a lycophyte. (A)** LOGO representation of the K-segment of non-vascular dehydrin sequences. **(B)** LOGO representation of a subset of non-vascular dehydrin sequences. Blue – positively charged (Lys, Arg, His); red – negatively charged (Asp, Glu); black – hydrophobic (Ala, Val, Leu, Ile, Pro, Phe, Met), green – polar (Gly, Ser, Thr, Tyr, Cys), purple – neutral (Asn, Gln). **(C)** Dehydrin sequence from the lycophyte *Selaginella moellendorffii*. Residues are colored according to: acidic residues, red; basic residues, light blue; aromatic residues, dark blue; hydrophobic residues, magenta; polar residues, brown; Pro or Gly, orange; Cys, green. The K-segment is boxed and labeled.

In non-vascular plants, the K-segment has been suggested to have weaker similarity to the K-segment found in vascular plants (Velten and Oliver, 2001; Saavedra et al., 2006). We therefore used a combined approach of four different methods to search for potential dehydrins in non-vascular embryophytes: (i) BLASTP search (Altschul et al., 1997) using previously identified *P. patens* dehydrins (Ruibal et al., 2012); (ii) BLASTP search using dehydrins identified in (i); (iii) MAST search (Bailey and Gribskov, 1998) using the vascular K-segment; (iv) MAST search using the non-vascular K-segment identified in (i). The resulting motif is shown in LOGO form in **Figure 10A**. Interestingly, the most prevalent amino acids appear to be similar to the vascular K-segment in **Figure 2A**, and different from the K-segments identified in *P. patens* (Saavedra et al., 2006) and a dehydrin-like protein in *Tortula ruralis* (Velten and Oliver, 2001).

At this point we made a visual inspection of the non-vascular dehydrins, and noted that there were two kinds – one that resembled the vascular K-segment, and another that contained a less conserved Lys and Glu/Asp residues (**Figure 10B**). We also realized that an analysis of the lycophyte *Selaginella moellendorffii* genome did not detect any dehydrins. We repeated the search using the K-segment found in **Figure 10B** and interestingly found one sequence, shown in **Figure 10C**, that matched the K-segment motif from the non-vascular plants.

Using the K-segment motif in **Figure 10B** we did not discover any new dehydrin sequences in the other vascular plant genomes. Note also that no matches were found in any of the seven green algae genomes that were analyzed (data not shown).

## Discussion

Our study of 643 vascular plant dehydrin sequences provides us with a detailed description of the conserved sequences in the K-, Y-, and S-segments (**Figures 2–4**). The motifs from previous dehydrin studies are similar to the MEME defined motifs, the comprehensive analysis performed here will help in identifying the key functional residues in this protein family. The oft-cited canonical K-segment (EKKGIMDKIKEKLPG) contains considerable variation. It is interesting to look closely not just at the conservation of the specific amino acid, but also the conservation of its chemical property. In addition to the conservation of numerous lysine residues, several positions in the K-segment show preservation of the hydrophobic character (**Figure 2**). We propose that these two conservations (positive charge and hydrophobicity) are important for peripheral membrane binding by the K-segment. Dehydrins are able to lower the transition temperature of a membrane (Eriksson et al., 2011), and in our own work we have observed that the K-segment is able to prevent membrane fusion (Clarke et al., 2015). The positively charged Lys residues are likely important for interacting with negatively charged membranes (Koag et al., 2003). Surprisingly, Arg, another positively charged amino acid, is found very infrequently in the K-segment (<5% at the key Lys positions of 2, 8, 10, and 12). Similarly, the His amino acid, with its weak positive charge at physiological pH, is present at <1% at the key Lys positions. Clearly there is an additional property at play other than just charge. One possibility is the ability of the lysine side chain to snorkel, that is, the methylene backbone of the side chain can interact with the aliphatic chains while the positively charged amide group “snorkels” to make contact with the negatively charged phosphate headgroup (Strandberg and Killian, 2003).

In the case of the Y-segment sequence, the central Tyr residue is mostly conserved in this motif (**Figure 3**). The other commonly occurring amino acids at this position are His and Phe, suggesting that it is the aromatic nature that is important at this position. While we think that free nucleotide binding is not a likely function of the Y-segment (reviewed in Graether and Boddington, 2014), the importance of the aromatic side chain suggests that this motif may be important for interacting with DNA through base stacking interactions. **Figure 3** also identifies the conservation of amino acid properties at the seventh position, which is dominated by hydrophobic amino acids (mostly Val or Ile), which was not previously identified as part of the Y-motif.

Another interesting conservation issue was seen for the S-segment, where despite the presence of a hydroxyl group, Thr residues are not found in the Ser tract. In this case it could be that Ser, as a secondary structure breaker, is more compatible with the intrinsically disordered nature of dehydrins compared to the β-strand promoting Thr (Smith et al., 1994). The motif search of the S-segment also revealed two conserved regions outside of the Ser tract: one that includes several residues to the N-terminal end of the Ser tract (**Figure 4**), and a second one that shows that some S-segments are closely linked to the K-segment (the SK-segment; **Figure 6C**). The N-terminal extension begins with Leu-His-Arg, where the His can be replaced with Arg or Gln (**Figure 4A**). Other studies have suggested that the extended S-segment in dehydrins contains signals for phosphorylation by two different enzymes: casein kinase II (CKII; Jensen et al., 1998) and Snf1-related kinase (Snf1RK; Vlad et al., 2008). In the case of CKII, the consensus signal is the acidic residues located after the Ser tract, while in the case of Snf1RK it is LXRXXS. It has also been suggested that multiple kinases are required to phosphorylate the S-segment, since the *in vitro* phosphorylation level achieved by CKII or Snf1RK alone is not the same as that detected *in vivo* (Jiang and Wang, 2004; Alsheikh et al., 2005). Our sequence analysis supports the idea that these multiple signals may promote phosphorylation by multiple kinases (Alsheikh et al., 2005), however, it is not yet clear if multiple phosphorylation events occur on a single protein. While the phosphorylated S-segment has potential roles in nuclear localization (Goday et al., 1994; Jensen et al., 1998) and/or calcium binding (Alsheikh et al., 2003), the need for multiple kinases has not yet been explained. Two possibilities include that the dehydrins evolved this way in order to deal with the multiple Ser residues, or that the multiple phosphorylation signals serve as some kind of a consensus signal (Rust and Thompson, 2011), perhaps as a measure of the cellular stress level.

The detection of the SK-segment came about from an examination of the distances between the S- and K-segments (**Figure 6B**), where a very short distance of 3–6 residues was observed more than half of the time. A motif analysis of this region revealed that the two segments were often separated by a Gly-Xaa-Gly-Gly motif and the K-segment often began with the Arg-Arg-Lys-Lys motif (**Figure 6C**). The GXGG motif would be a highly flexible segment, even in a disordered protein. We speculate that the SK-segment arrangement may be involved in regulating transport of a dehydrin into the nucleus. Previous studies have shown that the S-segment can be phosphorylated, and that phosphorylation of this motif is required for nuclear localization (Goday et al., 1994). The proximity of a phosphorylated S-segment to the K-segment, and its connection by a highly flexible GXGG motif, could allow the negatively charged phosphoserines to interact with the K-segment, while the RRKK motif has been suggested to also be a nuclear localization signal (Kalderon et al., 1984; Jensen et al., 1998). This binding could have multiple effects: to reduce the K-segment’s affinity for the membranes, allowing it to diffuse into the cytoplasm and eventually nucleus; to reduce the relatively large hydrodynamic radius of the protein so that it is smaller and could cross the nuclear pore complex; and possibly to expose the RRKK motif so that it could act as a better nuclear localization signal.

We sought to find associations between dehydrin architectures and the ability of a particular plant species to withstand one of the three major abiotic stresses (drought, cold, or salinity). Many previous studies have suggested that certain dehydrin architectures may be used to protect from certain abiotic stresses (reviewed in Graether and Boddington, 2014). A summary of the plant’s relative ability to withstand these stresses and the dehydrin’s architecture is summarized in **Table 4**. The table does not show any obvious systematic pattern between stress resistances and protein architecture, but it does not take into account the different biochemical properties within a particular architecture. The examination of dehydrin gene expression by architecture (**Figure 9**) shows that individual levels of expression can vary considerably between individual dehydrins even with the same architecture. While the anatomy and developmental stages (**Figures 9B,C**) poorly correlate with architecture, the architectures do appear to have some preference for the different abiotic stresses (**Figure 9A**). For the most part, these patterns are similar to what we have previously suggested (Graether and Boddington, 2014), with the exception being that K_n_ dehydrins appear to be important during drought and not just cold. It must be noted that different plants have evolved different mechanisms to adapt to the cold, drought, and salinity (Ahmad and Prasad, 2012), and that dehydrins represent only a part of the response.

**Table 4:** Ability of a plant species to withstand an abiotic stress and its association with dehydrin architecture.

	Stress			Architecture		
Organism	Drought	Cold	Salinity	K_n_	Y_n_SK_n_	Y_n_K_n_	SK_n_	K_n_S	Total
*M. truncatula*	+	–	+	1	1	0	2	2	6
*P. vulgaris*	–	–	–	1	1	1	1	0	4
*G. max*	0	0	0	1	1	3	1	1	7
*C. sativus*	0	0	0	1	1	0	1	0	3
*P. persica*	0	0	–	0	2	2	2	0	6
*M. domestica*	0	+	–	5	4	1	5	0	15
*F. vesca*	–	+	–	0	5	0	1	0	6
*A. thaliana*	+	+	–	1	2	1	5	1	10
*A. lyrata*	+	+	–	4	2	1	2	1	10
*C. rubella*	–	0	0	2	2	0	4	1	9
*C. grandiflora*	–	0	0	2	0	0	5	1	8
*B. stricta*	–	+	–	3	2	0	3	1	9
*B. rapa*	–	+	0	1	4	1	5	1	12
*E. salsugineum*	0	+	+	0	3	0	4	1	8
*C. papaya*	–	–	0	0	2	0	1	1	4
*G. raimondii*	+	–	+	1	2	1	3	0	7
*T. cacao*	–	–	–	1	2	0	1	1	5
*C. sinensis*	0	+	–	2	2	1	1	2	8
*C. clementine*	0	–	–	1	1	0	1	0	3
*M. esculenta*	–	0	–	0	2	0	1	2	5
*R. communis*	+	–	0	0	2	1	1	1	5
*L. usitatissimum*	–	+	–	2	2	2	4	1	11
*P. trichocarpa*	–	+	–	3	0	1	1	1	6
*E. grandis*	–	0	–-	2	2	0	1	0	5
*V. vinifera*	–	0	–	0	0	1	1	0	2
*S. tuberosum*	0	0	0	1	2	1	1	0	5
*S. lycopersicum*	–	–	0	1	3	0	2	0	6
*M. guttatus*	–	+	–	0	2	0	1	1	4
*A. coerulea*	–	+	–	1	1	0	2	1	5
*S. bicolor*	+	–	0	0	3	0	1	1	5
*Z. mays*	–	–	–	0	3	0	2	2	7
*S. italica*	–	+	–	0	5	0	1	0	6
*P. virgatum*	0	+	0	0	9	0	3	1	13
*O. sativa*	–	–	0	0	6	0	1	1	8
*B. distachyon*	+	+	0	0	5	0	4	1	10

*The resistance ratings were classified into three ranges: good (+), moderate (0), or weak (-). Classifications were obtained from several sources (http://plants.usda.gov/java/characteristics, http://www.fao.org/ag/AGp/agpc/doc/gbase/latinsearch.htm, http://hort.ifas.ufl.edu/database/trees/trees_scientific.shtml, http://dgnurseries.com/product-category/native-plants/, http://phytozome.jgi.doe.gov).*

A key technique in the dissection of protein function is the mutation of potentially important residues or stretches of residues. The lack of strict sequence conservation in IDPs like dehydrins compared to well-ordered proteins makes this a challenge. A common technique, such as alanine scanning, may not be the best choice since the hydrophobic nature of this amino acid and its propensity to from α-helices could change the dehydrin’s properties in undesired ways. The position-weighted matrices for the various segments (**Figures 2B, 3B, 4B**) can be used to guide mutation decisions that help to ensure that residue changes are made to amino acids that are never or very rarely found at that position, while still choosing ones that are not likely to induce structure or change the hydrophilic character of dehydrins. Deletion mutations in dehydrin genes must also be made with care. The overall length of the dehydrin can be another important property to conserve, as was shown in the cryoprotective activity of a dehydrins (Hughes et al., 2013).

Our study shows that the five dehydrin architectures (K_n_, Y_n_SK_n_, Y_n_K_n_, SK_n_, or K_n_S) still hold true for nearly all dehydrins from vascular plants. We also provide position-weighted matrices for searching for the K-, Y-, and S-segments that can be applied in the search for novel dehydrins. Additionally, we found that the Y-segment can often contain a phenylalanine or histidine instead of the central tyrosine, and that in almost all Y_n_SK_n_ dehydrins the S-segment and K-segments are linked by a short span of residues, suggesting that they may play some structure/function role. These results provide important guidelines for creating dehydrin mutants that do not inadvertently cause unwanted alterations, such as a gain in structures or failing to maintain an important property (e.g., length or charge).

## Author Contributions

All authors contributed to the design of the experiments and the writing of the manuscript. AM, MV, and SG: performed the bioinformatic sequence analysis. KB: assembled and analyzed the relationship between the abiotic stress and the dehydrin architectures. KS: assembled and analyzed the relationship between the dehydrin gene upregulation and the various conditions.

## Conflict of Interest Statement

The authors declare that the research was conducted in the absence of any commercial or financial relationships that could be construed as a potential conflict of interest.

## Supplementary Material

FILE S1 **List of all dehydrins discovered in Phytozome 10**.
