# Supplementary material for: Genome Analysis of Conserved Dehydrin Motifs in Vascular Plants
Source: Front Plant Sci. 2017 May 4;8:709. doi: 10.3389/fpls.2017.00709 (PMC5415607; doi:10.3389/fpls.2017.00709)
Supplement: Supplementary file 2 [file Table_1.DOCX]

**Table S1. Definition of the K-segment from the literature.**

| **Authors & year** | **Sequence** |
| --- | --- |
| (Close et al., 1989) | EKKG[I/V]DKIKEKLPG  RKKG[M/I/L]K[E/D]KIKEKLPG |
| (Close, 1996) | [EHQK]K[KE][GS][IFMVL][MLVAT][DEG]KIK[EDQ][KQ][LIF][PHT]G |
| (Artlip et al., 1997) | \| EKKGIMDKIKEKLPG \| \| --- \| \| E[KR]KG[ILV][MLIVG][DGE][KQ][ILV][KQE][ED]KLPG \| |
| (Campbell and Close, 1997) | [EHQKD]K[KE][GS][IFMVL][MLVAIT][DEG]K[IV]K[EDQ][KQ][LIF][PHTL]G  [Q/E]K[P/A]G[M/L]LDKIK[A/Q][K/M][I/L]PG |
| (Allagulova et al., 2003) | [Q/E]K[P/A]G[M/L]LDKIK[A/Q][K/M] [I/L]PG |
| (Garcia-Bañuelos et al., 2009) | RKKGLKEKIKEKLPG  TTYKVGEQRQEKLPG  QEKGTTDKIKEKLPG  EKKNMMDKIKEKLPG  EKKGIMDKIKEKLPG |
| (Eriksson et al., 2011) | EKKGIMDKIKEKLPG  RKKGIKEKIKEKLPG  GEKKGIMDKIKEKLPG  [DEGHKNQR][GKNR][DEGKQ][AGNSW][AFILMTV][AFGIKLMTV]  [DEGNQY][KNQR][ILNV][KMQ][DE][KNQT][FILM][HPS][GL]  [EGHKQR][DGKNR][EGKQ][ADGKSW][AFGILMSTV][AFGILFMTV]  [DEFGLMNQV][DEGKNQR][IKLNV][IKMQ][DEK][DEKNQT][FIKL]  [HILMPS][GLP] |
| (Yang et al., 2012) | [QE][QK]KG[MI][ML]EKIK[ED]KLPG  [RE]KKG[MLF][KL][ED]KIKE[RK][IL][PT]G |
| (Du et al., 2013) | EKKG[IL][IM][EDG][KQ][IV]K[ED]KLPG |
| (Omar et al., 2013) | KKKGLKEKIKEKLTG  EKKSVMEKIKEKLPG |
