# Supplementary material for: Genome Analysis of Conserved Dehydrin Motifs in Vascular Plants
Source: Front Plant Sci. 2017 May 4;8:709. doi: 10.3389/fpls.2017.00709 (PMC5415607; doi:10.3389/fpls.2017.00709)
Supplement: Supplementary file 3 [file Table_2.DOCX]

| Variable | Low | Medium | High |
| --- | --- | --- | --- |
| pI | <6.5 | 6.5 - 7.5 | >7.5 |
| M_r_ | <15 kDa | 15-25 kDa | >25 kDa |
| GRAVY score | <-1.4 | -1.4 to -1.0 | >-1.0 |

**Table S2. CATPCA variable classifications**
