# Supplementary material for: Genome Analysis of Conserved Dehydrin Motifs in Vascular Plants
Source: Front Plant Sci. 2017 May 4;8:709. doi: 10.3389/fpls.2017.00709 (PMC5415607; doi:10.3389/fpls.2017.00709)
Supplement: Supplementary file 4 [file Table_3.DOCX]

| Species | K_n_ | Y_n_SK_n_ | Y_n_K_n_ | SK_n_ | K_n_S | SK | Acidic  pI | Neutral  pI | Basic  pI | Low  MW | Medium  MW | High  MW | Low  GRAVY | Medium  GRAVY | High  GRAVY |
| --- | --- | --- | --- | --- | --- | --- | --- | --- | --- | --- | --- | --- | --- | --- | --- |
| *M. truncatula* | 1 | 1 | 0 | 1 | 2 | 0 | 2 | 2 | 1 | 3 | 2 | 0 | 3 | 0 | 2 |
| *P. vulgaris* | 1 | 1 | 1 | 1 | 0 | 1 | 3 | 0 | 1 | 1 | 2 | 1 | 1 | 2 | 1 |
| *G. max* | 2 | 1 | 2 | 1 | 1 | 1 | 5 | 1 | 1 | 2 | 3 | 2 | 2 | 4 | 1 |
| *C. sativus* | 1 | 1 | 0 | 2 | 0 | 0 | 2 | 1 | 1 | 1 | 2 | 1 | 3 | 1 | 0 |
| *P. persica* | 0 | 2 | 2 | 2 | 0 | 2 | 5 | 0 | 1 | 0 | 3 | 3 | 2 | 3 | 1 |
| *M. domestica* | 5 | 3 | 1 | 5 | 0 | 4 | 4 | 2 | 8 | 3 | 5 | 6 | 5 | 6 | 3 |
| *F. vesca* | 0 | 5 | 0 | 1 | 0 | 4 | 1 | 2 | 3 | 0 | 2 | 4 | 1 | 4 | 1 |
| *A. thaliana* | 3 | 3 | 1 | 5 | 1 | 2 | 8 | 2 | 3 | 2 | 8 | 3 | 2 | 10 | 1 |
| *A. lyrata* | 4 | 2 | 1 | 2 | 1 | 2 | 6 | 1 | 3 | 2 | 6 | 2 | 3 | 5 | 2 |
| *C. rubella* | 2 | 2 | 0 | 4 | 1 | 2 | 4 | 3 | 2 | 2 | 5 | 2 | 3 | 5 | 1 |
| *C. grandiflora* | 2 | 0 | 0 | 5 | 1 | 2 | 4 | 1 | 3 | 3 | 3 | 2 | 3 | 5 | 0 |
| *B. stricta* | 3 | 2 | 0 | 3 | 1 | 2 | 4 | 3 | 2 | 2 | 5 | 2 | 4 | 5 | 0 |
| *B. rapa* | 1 | 4 | 1 | 5 | 1 | 4 | 7 | 3 | 2 | 2 | 8 | 2 | 4 | 6 | 2 |
| *E. salsugineum* | 0 | 3 | 0 | 4 | 1 | 2 | 4 | 3 | 1 | 2 | 4 | 2 | 2 | 4 | 2 |
| *C. papaya* | 0 | 2 | 0 | 1 | 1 | 2 | 2 | 1 | 1 | 2 | 2 | 0 | 2 | 2 | 0 |
| *G. raimondii* | 4 | 2 | 1 | 5 | 0 | 1 | 10 | 0 | 2 | 3 | 9 | 0 | 8 | 4 | 0 |
| *T. cacao* | 1 | 2 | 0 | 1 | 1 | 0 | 2 | 2 | 1 | 1 | 3 | 1 | 2 | 3 | 0 |
| *C. sinensis* | 2 | 2 | 1 | 1 | 0 | 0 | 3 | 1 | 2 | 1 | 4 | 1 | 2 | 3 | 1 |
| *C. clementine* | 1 | 1 | 0 | 1 | 0 | 1 | 2 | 0 | 1 | 0 | 2 | 1 | 1 | 2 | 0 |
| *M. esculenta* | 0 | 2 | 0 | 1 | 2 | 2 | 2 | 2 | 1 | 2 | 3 | 0 | 4 | 1 | 0 |
| *R. communis* | 0 | 2 | 1 | 1 | 1 | 5 | 2 | 2 | 1 | 1 | 4 | 1 | 3 | 2 | 0 |
| *L. usitatissimum* | 2 | 2 | 2 | 4 | 1 | 2 | 8 | 1 | 2 | 1 | 8 | 2 | 1 | 8 | 2 |
| *P. trichocarpa* | 1 | 0 | 1 | 1 | 1 | 0 | 3 | 0 | 2 | 0 | 1 | 3 | 1 | 3 | 0 |
| *E. grandis* | 2 | 2 | 0 | 1 | 0 | 3 | 3 | 0 | 2 | 2 | 3 | 0 | 1 | 4 | 0 |
| *V. vinifera* | 0 | 0 | 1 | 1 | 0 | 2 | 1 | 0 | 1 | 1 | 1 | 0 | 1 | 0 | 1 |
| *S. tuberosum* | 1 | 2 | 1 | 1 | 0 | 3 | 2 | 3 | 0 | 2 | 2 | 1 | 1 | 3 | 1 |
| *S. lycopersicum* | 2 | 3 | 0 | 1 | 3 | 4 | 2 | 2 | 2 | 3 | 2 | 1 | 2 | 2 | 2 |
| *M. guttatus* | 0 | 2 | 0 | 0 | 1 | 0 | 1 | 2 | 0 | 1 | 1 | 1 | 1 | 2 | 0 |
| *A. coerulea* | 1 | 1 | 0 | 2 | 1 | 1 | 3 | 0 | 2 | 1 | 2 | 2 | 3 | 1 | 1 |
| *S. bicolor* | 0 | 2 | 0 | 1 | 1 | 0 | 1 | 1 | 2 | 1 | 1 | 2 | 1 | 2 | 1 |
| *Z. mays* | 0 | 3 | 0 | 2 | 3 | 3 | 3 | 1 | 3 | 1 | 2 | 4 | 1 | 4 | 2 |
| *S. italica* | 0 | 6 | 0 | 1 | 0 | 4 | 3 | 0 | 4 | 2 | 1 | 4 | 0 | 4 | 3 |
| *P. virgatum* | 0 | 7 | 0 | 4 | 1 | 1 | 3 | 3 | 6 | 5 | 3 | 4 | 1 | 8 | 3 |
| *O. sativa* | 0 | 6 | 0 | 1 | 1 | 6 | 1 | 2 | 5 | 1 | 4 | 3 | 1 | 5 | 2 |
| *B. distachyon* | 3 | 2 | 0 | 3 | 1 | 7 | 3 | 1 | 6 | 1 | 1 | 3 | 1 | 6 | 3 |

**Supplemental Table S3. CATPCA variable inputs.**
