# Supplementary material for: Genome Analysis of Conserved Dehydrin Motifs in Vascular Plants
Source: Front Plant Sci. 2017 May 4;8:709. doi: 10.3389/fpls.2017.00709 (PMC5415607; doi:10.3389/fpls.2017.00709)
Supplement: Supplementary file 5 [file Table_4.DOCX]

**Table S4.** MEME parameters used in the motif search

| Search | Width | Max. dataset size | Max. number  of motifs | Number of motifs |
| --- | --- | --- | --- | --- |
| K-segment | 15 | 70000 | 3000 | 1 |
| Y-segment | 7 | 70000 | 1500 | 4 |
| S-segment | 7-20 | 70000 | 3000 | 10 |
| Motif discovery | 8-50 | 100000 | 5000 | 10 |
| GT-motif | 5-20 | 70000 | 3000 | 10 |
| ^S^K-motif | 40 | 70000 | 3000 | 3 |
