# Supplementary material for: Genome Analysis of Conserved Dehydrin Motifs in Vascular Plants
Source: Front Plant Sci. 2017 May 4;8:709. doi: 10.3389/fpls.2017.00709 (PMC5415607; doi:10.3389/fpls.2017.00709)
Supplement: Supplementary file 6 [file Table_5.DOCX]

**Table S5.** Developmental stage groupings

| General Developmental Stage | Specific Developmental Stages |
| --- | --- |
| Early | Germination  Seedling Growth  Young Rossette  Inflorescence Visible |
| Middle | Developed Rossette  Bolting  Young Flower  Stem Elongation  Inflorescence Formation Anthesis  Flowering  Fruit Formation  Tillering  Booting  Heading  Axillary Shoot Formation  Flower Emergence  Main Shoot Growth Inflorescence Emergence  Flowers and Siliques  Main Shoot Growth |
| Late | Developed Flower  Mature Siliques  Dough Development  Ripening  Fruit Ripening Complete  Milk Development  Flowers and Pods  Mature Pods  Senescence |
