# Supplementary material for: Genome Analysis of Conserved Dehydrin Motifs in Vascular Plants
Source: Front Plant Sci. 2017 May 4;8:709. doi: 10.3389/fpls.2017.00709 (PMC5415607; doi:10.3389/fpls.2017.00709)
Supplement: Supplementary file 7 [file Image_1.PDF]

Fig. S1

A (\wKKG\w{5,7}K\w[PSTH]G)
|(\w{7}K[FILMVA]K\wK\w?\w?[PSTH]G)
|(\w{7}K[FILMVA]K\w?\w?K\w{5})

B (DE\w?\w?\wNP)|(\w\w\wGNP)|(DEYG\w\w)

C ([LIHF]?[HRQET]?[RGH]?[ST]?\w?S?[SG]?S{4}
[VTN]?[DESK]?[DES]?[DESGF]?[EDSG]?[GEDQ])

Figure S1. Motif search definition for the dehydrin segments. The search strings are shown in the Perl regular expression format. A) K-segment. B) Y-segment. C) S-segment.
