# Supplementary material for: Genome Analysis of Conserved Dehydrin Motifs in Vascular Plants
Source: Front Plant Sci. 2017 May 4;8:709. doi: 10.3389/fpls.2017.00709 (PMC5415607; doi:10.3389/fpls.2017.00709)
Supplement: Supplementary file 8 [file Image_2.PDF]

Fig. S2

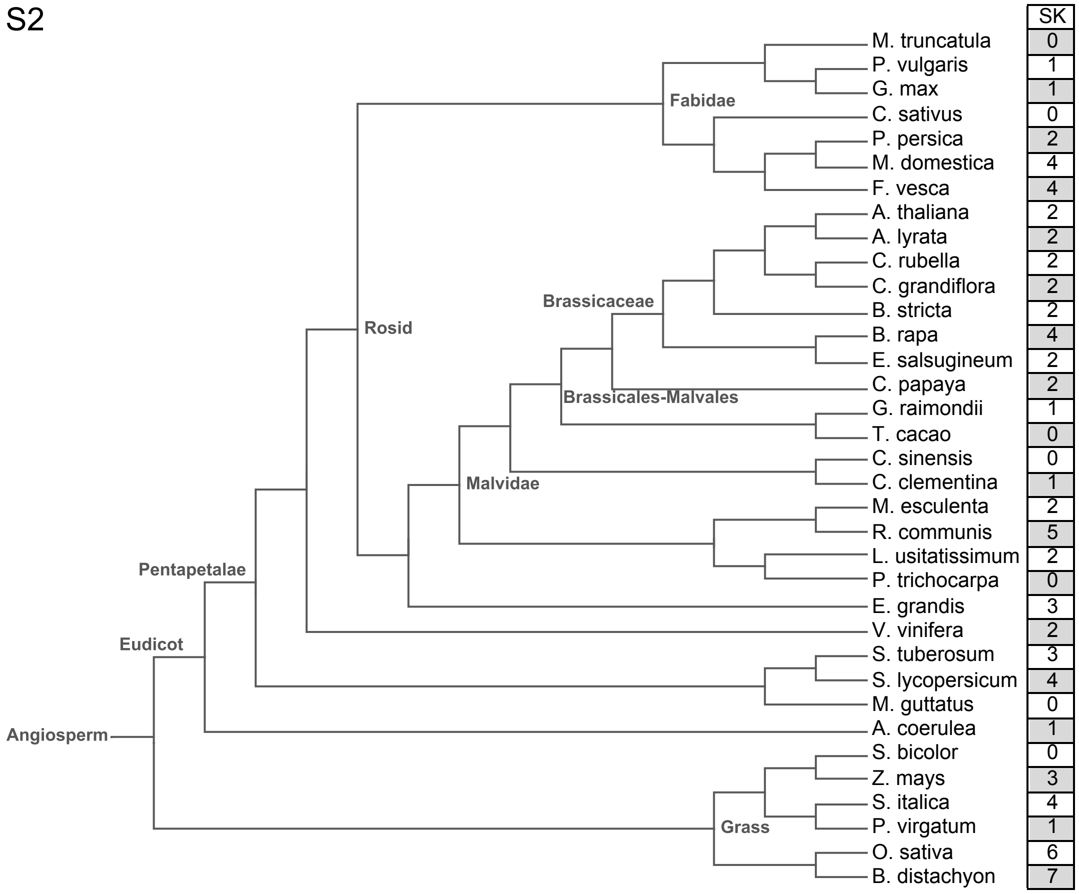

Figure S2. Distribution of the SK-segment by species. A phylogenetic tree of the species from Phytozome 10 used in this study. The right column indicates the count of the SK-segment found in each species.
